# *Teratorn* and its relatives – a cross-point of distinct mobile elements, transposons and viruses

**DOI:** 10.3389/fvets.2023.1158023

**Published:** 2023-04-28

**Authors:** Yusuke Inoue, Hiroyuki Takeda

**Affiliations:** Department of Biological Sciences, Graduate School of Science, The University of Tokyo, Tokyo, Japan

**Keywords:** mobile genetic element, transposable element, virus, *piggyBac*, herpesvirus, teleost, recombination

## Abstract

Mobile genetic elements (e.g., transposable elements and plasmids) and viruses display significant diversity with various life cycles, but how this diversity emerges remains obscure. We previously reported a novel and giant (180 kb long) mobile element, *Teratorn*, originally identified in the genome of medaka, *Oryzias latipes*. *Teratorn* is a composite DNA transposon created by a fusion of a *piggyBac*-like DNA transposon (*piggyBac*) and a novel herpesvirus of the *Alloherpesviridae* family. Genomic survey revealed that *Teratorn*-like herpesviruses are widely distributed among teleost genomes, the majority of which are also fused with *piggyBac,* suggesting that fusion with *piggyBac* is a trigger for the life-cycle shift of authentic herpesviruses to an intragenomic parasite. Thus, *Teratorn*-like herpesvirus provides a clear example of how novel mobile elements emerge, that is to say, the creation of diversity. In this review, we discuss the unique sequence and life-cycle characteristics of *Teratorn*, followed by the evolutionary process of *piggyBac*-herpesvirus fusion based on the distribution of *Teratorn*-like herpesviruses (relatives) among teleosts. Finally, we provide other examples of evolutionary associations between different classes of elements and propose that recombination could be a driving force generating novel mobile elements.

## Introduction

Cellular organisms have been parasitized by selfish-replicating genetic entities in the history of life, which include transposable elements (TE) and viruses, as well as plasmids and self-splicing elements (1). Viruses and mobile genetic elements (MGEs) are very abundant biological entities on earth; viruses are estimated to outnumber 10 to 100-fold relative to cellular organisms (2), while TEs often occupy a large part of the host genome [e.g., ~45% in human (3) and ~ 85% in maize (4)]. TEs and viruses frequently cause harmful effects on host organisms, such as insertional mutation by TEs and pathogenesis by viruses. To counter selfish elements, cellular organisms have developed various defense systems, such as RNA interference, epigenetic silencing, and innate and adaptive immune systems (5–7). On the other hand, host organisms have sometimes co-opted selfish elements for their own functions, such as novel gene regulatory networks by many TEs (8–10), V(D)J recombination system in vertebrates from a *Transib*-like transposon (11), polydnaviruses in parasitoid wasps (12) and placenta in mammals from retroviruses (8, 13, 14). Together, MGEs and viruses have been key players in the evolution of cellular organisms. Despite their importance, however, we are far from full understanding of how MGEs and viruses themselves have evolved; that is, their origin, relationships among different classes of elements, and underlying mechanisms of the emergence of novel elements. This is attributed to the enormous sequence diversity caused by the rapid evolution rate and frequent gene gain/loss (15).

MGEs and viruses are often classified based on their genome constitution (either DNA or RNA of double-stranded (ds) or single-stranded (ss) nucleotides), replication manner (e.g., DNA polymerization, RNA-dependent RNA transcription, reverse transcription), and life cycle (with/without extracellular phase) (1, 16, 17). Eukaryotic TEs are categorized into two classes, according to whether or not they include RNA intermediates. Class I elements (retrotransposons) include long terminal repeat (LTR) and non-LTR elements, both of which transpose in a “copy-and-paste” manner mediated by RNA transcription and reverse transcription. Class II elements (DNA transposons) are further classified into two subclasses, depending on the type of transposition; i.e., in a “cut-and-paste” (e.g., *P* element, *Tc1/mariner*, *hAT*, *piggyBac*) or “copy-and-paste” manner (e.g., *Helitron*, *Polintons*/*Mavericks*) (16). Similarly, eukaryotic viruses are classified into the following groups according to their genomic entities and replication manner; dsDNA viruses (e.g., Poxviruses, Herpesviruses, Adenoviruses, and Polyomaviruses), ssDNA viruses (e.g., Geminiviruses and Circoviruses), dsRNA viruses (e.g., Reoviruses), positive-strand ssRNA viruses (e.g., Picornaviruses, Coronaviruses, and Tombusviruses), negative-strand ssRNA viruses (e.g., Orthomyxoviruses and Filoviruses), ssRNA-reverse transcriptase (RT) viruses (e.g., Retroviruses) and dsDNA-RT viruses (e.g., Pararetroviruses) (17). Given their enormous diversity, as well as the absence of genes conserved among all elements, it had long remained unclear the evolutionary relationships among different classes of elements, except for a few well-known cases (e.g., ssRNA-RT viruses and LTR retrotransposons) (14, 18). However, recent advances in whole-genome shotgun sequencing technologies and metagenomic analyses have enabled us to identify many novel MGEs and viruses, providing evolutionary insights into the mechanisms underlying the emergence of distinct classes of elements.

We previously identified a giant (~180 kb long) DNA transposon, named *Teratorn*, in the genome of a small teleost fish medaka (*Oryzias latipes*) (19), which was the biggest TE ever found at that time. *Teratorn* is unique in that it contains the transposition machinery of a *piggyBac*-like DNA transposon including active transposase, as well as the whole genome of an intact herpesvirus genome. A comprehensive genomic survey indicated that *Teratorn*-like elements are widely distributed among several teleost genomes, some of which were shown to be a fused form, suggesting the generality of *piggyBac*-herpesvirus fusion (20, 21). We proposed that fusion with the DNA transposon caused a shift in life cycle of the herpesvirus to an intragenomic life form, enabling successful intragenomic propagation in teleosts.

Here we review the characteristics of *Teratorn* and its relative elements. First, we introduce the identification of *Teratorn* in the medaka genome. We then describe the sequence characteristics of *Teratorn* and discuss its uniqueness such as its large size for a transposon and life cycle. Finally, with a likely evolutionary scenario of *piggyBac*-herpesvirus fusion, we propose that recombination among different classes of elements could be a driving force for generating novel mobile elements.

### Identification of *Teratorn* from medaka spontaneous mutants

**Figure 1:**
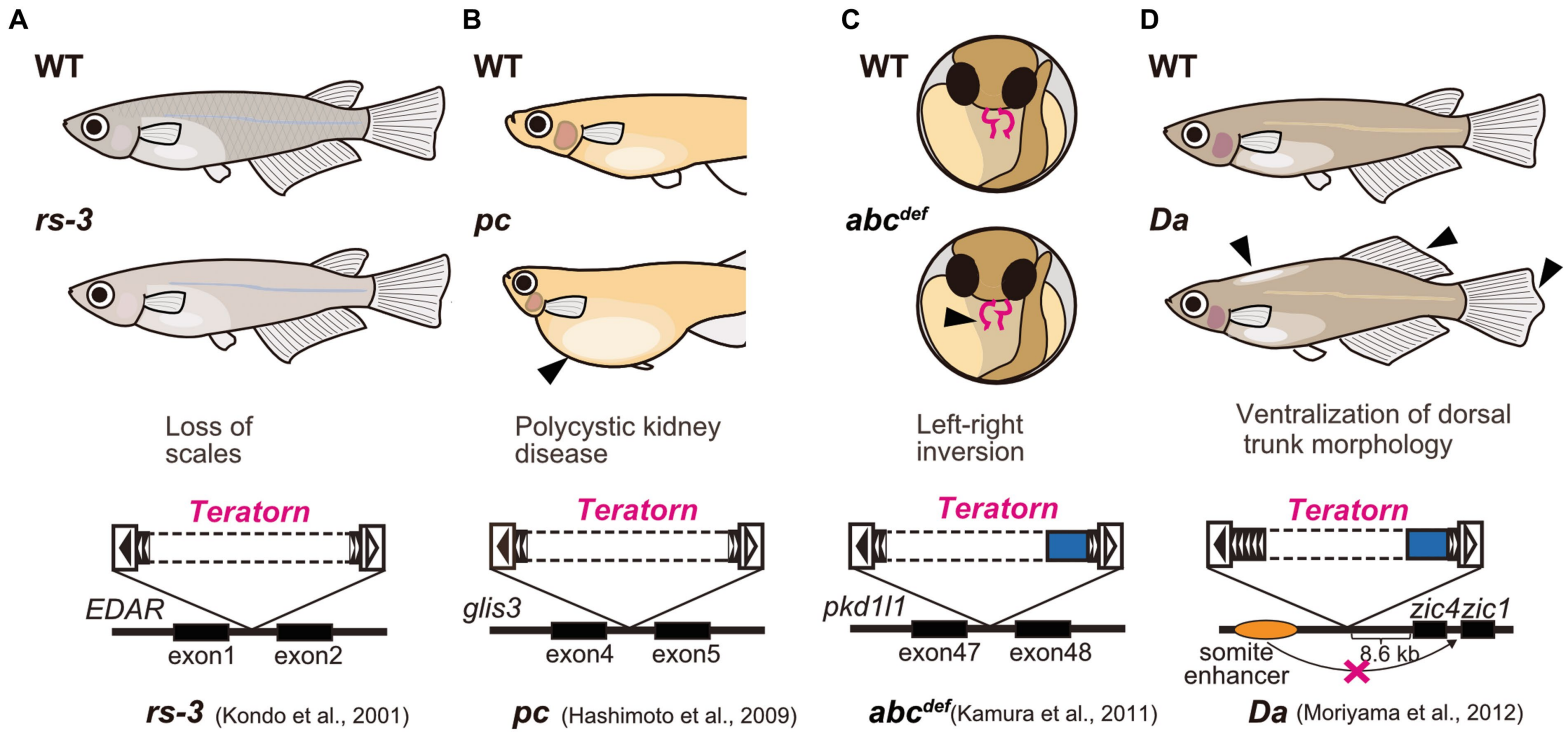
Medaka spontaneous mutants caused by *Teratorn* insertion. **(A)** *rs-3* mutant [loss of scales, (28)]. **(B)** *pc* mutant [polycystic kidney disease, (29)] **(C)** *abc^def^* mutant [defect in left–right axis formation, (30)]. **(D)** *Da* mutant [ventralization of dorsal trunk morphology, (31)]. Solid and dotted boxes inside *Teratorn* indicate the sequence-determined and undetermined region, respectively.

Medaka is a small egg-laying freshwater teleost fish that habitats in East Asia, including Japan, South Korea, Taiwan, and China. Medaka has long been utilized as an experimental vertebrate model organism along with zebrafish, because of transparent embryos, a short period of life cycle (2.5 to 3 months), high fecundity, easiness of genetic manipulation, and availability of the high-quality whole-genome sequencing data (22, 23). Furthermore, medaka has a strong advantage in the field of genetics in that in the long history of medaka research in Japan, various healthy inbred strains were established and various spontaneous mutants have been isolated [ref. Shimada and Takeda, 2010 (22)]. Importantly, active DNA transposons were isolated from medaka spontaneous mutants. For example, a series of genetic analyses of albino medaka mutants identified two active DNA transposons *Tol1* and *Tol2*, inserted at the tyrosinase gene (the rate-limiting enzyme for melanin biosynthesis) (24–26). These two elements belong to the *hAT* superfamily DNA transposons, are 4.4 kb and 4.7 kb long respectively, and encode the transposase and terminal inverted repeats (TIRs), the conventional form of DNA transposon that transpose in a cut-and-paste manner. These two elements were the first active DNA transposons reported in vertebrates (24, 25), and are currently widely utilized as a vector for transgenesis such as *Tol2*-mediated gene transfer in zebrafish (27). *Teratorn*, too, was originally isolated as causative DNA fragments for several medaka spontaneous mutants (Figure 1). First, Kondo et al. analyzed *rs-3* mutant, which shows almost complete loss of scales, and demonstrated that a transposon-like sequence (which later turned out to be *Teratorn*) was inserted in the first intron of ectodysplasin-A receptor (EDAR) (28) (Figure 1A). Similarly, Hashimoto et al. reported that the same sequence was inserted in the fourth intron of the *pc/glis3* gene, causing polycystic kidney disease (29) (Figure 1B). Our group also isolated the same insertions from two medaka spontaneous mutants; *abc^def^* [(30)] and *Da* [(31)]. In *abc^def^*, the DNA fragment is inserted at the 47^th^ intron of the *pkd1l1* gene, causing randomized left–right organ placement (30) (Figure 1C), while in *Da*, it is inserted in the regulatory region of *zic1*/*zic4* gene. The latter insertion impairs one of the tissue-specific enhancers of *zic1*/*zic4* gene, which results in specific loss of their expression in somites with the expression in the neural tube maintained, leading to ventralization of the dorsal part of the trunk morphology (e.g., fin morphology, pigmentation pattern, lateral line distribution, and body shape; Figure 1D) (31, 32). In the initial study, sequencing of fosmid clones at the *zic1/zic4* locus was performed and partial sequences of *Teratorn* were identified (about 20 kb long regions at both ends) (31). From the sequence information, we assumed that *Teratorn* is a DNA transposon that moves in a “cut-and-paste” manner, since it contains 18 bp terminal inverted repeats (TIR) at its termini, and is flanked by target site duplications (TSD) (16). Notably, this anticipated transposon was unique in size (at least 41 kb long) and in gene number (at least six genes), since most cut-and-paste DNA transposons are small (less than 10 kb long) and encode a limited number of genes (transposase) (31). We named this transposon *Teratorn* after the largest bird of prey that inhabited the North American continent. However, its complete sequence failed to be provided in the initial study, as it could not be deduced from the previously published medaka draft genome; the medaka genome has multicopy of the sequence as repetitive ones [ref. Kasahara et al., 2007 (33)].

### *Teratorn*: a 180 kb long giant DNA transposon created by a fusion of a *piggyBac*-like DNA transposon and a herpesvirus

**Figure 2:**
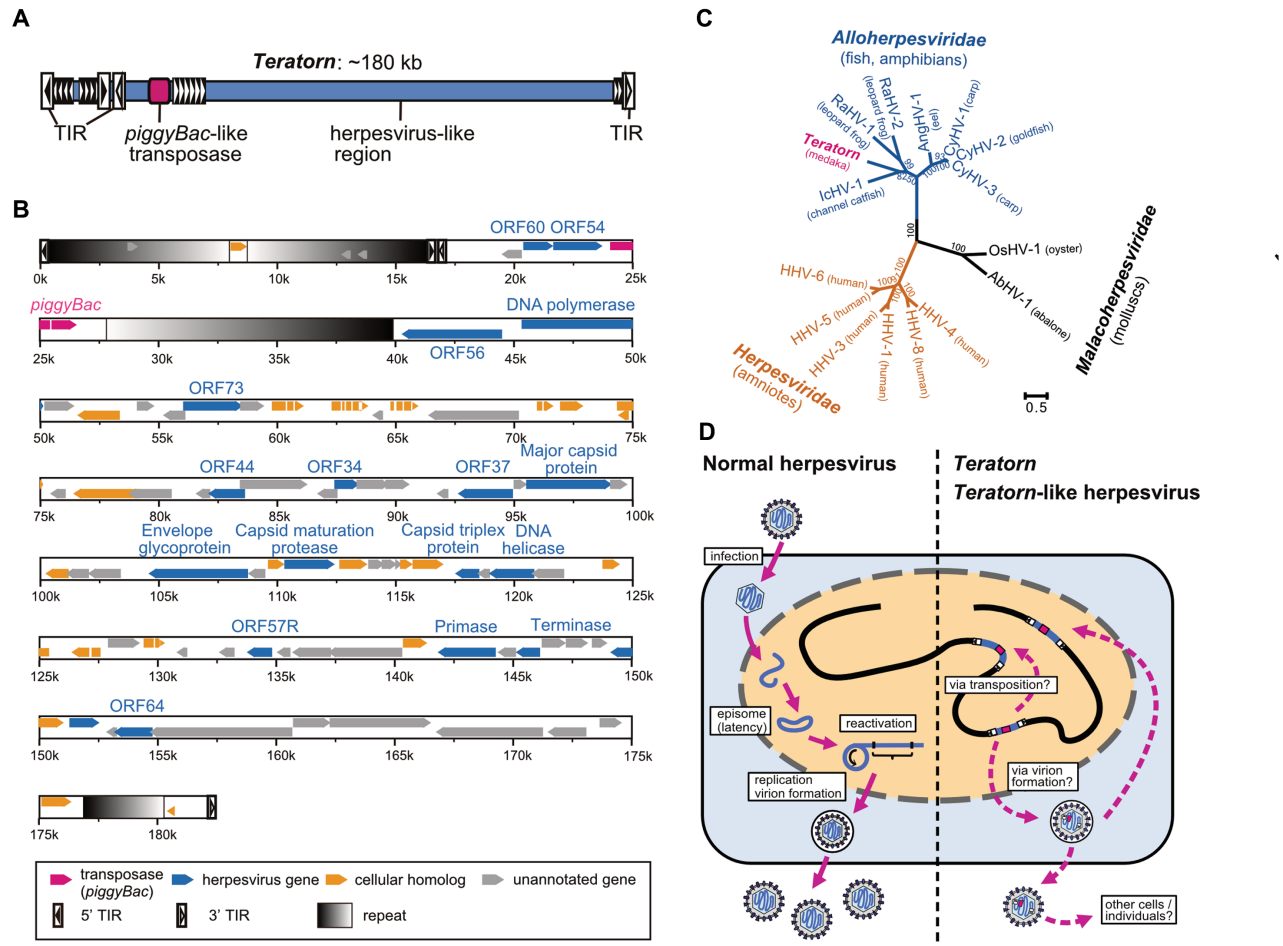
Sequence characteristics of *Teratorn* and its proposed life cycle. **(A)** The overall structure of *Teratorn*. **(B)** Gene map of the subtype 1 *Teratorn* copy. Predicted genes are classified into four categories depicted by colored arrowheads; magenta, *PiggyBac*-like transposase gene; blue, herpesvirus-like genes; yellow, cellular homologs; gray, unannotated genes. Terminal inverted repeats (TIRs) of *PiggyBac*-like transposon are depicted by squares with black-to-white gradients. **(C)** Phylogeny of *Teratorn* and representative herpesvirus species, based on the maximum-likelihood analysis. Amino acid sequences of the DNA packaging terminase, the only gene confidently conserved among all herpesvirus species, were used. Bootstrap values of branching are indicated at the nodes. **(D)** Comparison of life cycles of normal herpesviruses (left) and *Teratorn* (right).

To determine the full-length sequence of *Teratorn*, we screened a BAC library of Hd-rR strain to obtain clones containing the entire *Teratorn*. Using PacBio long-read sequencing, we found that *Teratorn* was ~180 kb long, quite larger than any other transposon reported at that time (Figure 2A). Further genomic survey indicated that there are two subtypes of *Teratorn*, which show ~88% sequence identity with each other, and their copy numbers were predicted to be 30 to 40 copies for subtype 1 and ~ 5 for subtype 2 in the Hd-rR strain medaka genome. Gene annotation inside *Teratorn* revealed two important points. First, a transposase gene, which is homologous to that of *piggyBac* superfamily DNA transposon, was identified (Figure 2B, red). *piggyBac* is one of the major “cut-and-paste” DNA transposon superfamilies and is widely distributed among eukaryotes, especially in metazoans (11). In addition, the sequence composition of terminal inverted repeats (TIRs) and target site duplications (TSDs) at its termini follows the rule of the *piggyBac* superfamily; they possess TIRs, 12–19 bp in length beginning with a “CCYT” motif, and preferentially target TTAA motif to give rise to TSDs. Furthermore, we experimentally showed that *Teratorn* transposase retains the transposition activity as demonstrated by *in vitro* assay with human culture cells; i.e., recognition of the TIRs, excision, and re-integration of the internal DNA sequence into chromosomal DNA. These characteristics indicate that *Teratorn* belongs to *piggyBac* superfamily and is still active as a transposon. Secondly, and quite surprisingly, besides the transposase gene, *Teratorn* encodes at least ~90 putative genes, 17 of which show sequence similarity to those of herpesviruses. Those include genes essential for herpesvirus life cycles, such as DNA polymerase, primase, UL21 homolog DNA helicase, capsid maturation protease, DNA packaging terminase, major capsid protein (MCP), subunit 2 capsid triplex protein, and envelope glycoprotein (Figure 2B, blue). Herpesviruses are double-stranded DNA viruses that infect a wide variety of vertebrate species and some molluscan species. Their genomes are relatively large, ranging from 124 to 295 kb, and contain 70 to 200 genes (34, 35). Phylogenetic analyses indicate that *Teratorn* belongs to *Alloherpesviridae* (infecting fish and amphibians), one of the three families of the order *Herpesvirales* (Figure 2C). Alloherpesviruses are known as several important pathogens in the field of aquaculture. Those include Cyprinid herpesvirus 3 (CyHV-3, known as koi herpesvirus) and Ictalurid herpesvirus 1 (IcHV-1, known as channel catfish virus), which caused massive outbreaks in the past (35). The existence of all 13 genes conserved among all alloherpesvirus species, as well as its size (~180 kb), strongly suggests that *Teratorn* contains the whole genome of a herpesvirus. Besides the essential genes, ~20 genes show no sequence similarity to those of other herpesviruses but are found in other organisms (Figure 2B, yellow). Most of them appear to function in regulation of immune response or cell proliferation in hosts. Given that alloherpesviruses primarily infect epithelial cells and cause pathologies such as epidermal cell necrosis, hyperplasia and lesions, branchial hyperplasia, papillomas and renal adenocarcinomas (35, 36), those genes could be utilized for the infection processes. Taken together, *Teratorn* is a complete fusion of a *piggyBac*-like DNA transposon and a whole herpesvirus genome.

### Giant TEs in eukaryotes; unique to *Teratorn*, or broadly existed?

One of the unique characteristics of *Teratorn* is its large size as compared with other transposons, since eukaryotic TEs are generally small (less than 10 kb long) and encode only a few genes (11, 16, 37). The exceptions to this rule were the *Ty3/Gypsy* family LTR retrotransposons *Ogre* in pea (~25 kb) (38) and *Burro* in the planarian (exceeding 30 kb) (39), Ac transposons in maize (up to 22 kb) (40–42), *Penelope*-like retrotransposons *Terminons* in rotifers (up to 40 kb) (43), *Helitrons* (up to 40 kb) (44, 45), and *Polintons/Mavericks* (up to 40 kb) (46, 47). However, TEs which exceed 50 kb were not reported until *Teratorn* was identified. One of the reasons why a few cases of large TEs had been reported could be the size limitation of TEs; the efficiency of reverse-transcription (for retrotransposons) and transposition (for cut-and-paste DNA transposons) decreases as its size increases (48). *Teratorn* could overcome the size limitation by acquiring the replication machinery of a herpesvirus genome (discussed later). The other reason could be technical difficulties in identifying large TEs from the draft genome, since repetitive sequences were hardly assembled from the whole-genome short-read data. However, recent advances in long-read sequencing technologies, as well as the wealth of genomic data of closely related species and strains, allow us to identify large TEs. For example, a series of studies reported a novel family of giant TEs, named *Starships* (reaching ~400 kb in size), in fungal genomes (49–51). Initially, members of the *Starship* elements were independently identified by several groups as sequences associated with specific traits of the hosts. Those include *HEPHAESTUS*, a ~ 85 kb long element containing many genes involved in tolerance to metal ions in *Paecilomyces vcariotii* (50), and *Enterprise*, reaching up to 247 kb, some of which contain a meiotic drive gene of the *Spoks* (spore killing) (51). A comprehensive genomic survey across *Ascomycetes* fungi genomes was later performed and proposed that *HEPHAESTUS* and *Enterprise* belong to the same family of giant mobile element *Starships* (49). *Starships* share characteristic genes such as tyrosine recombinase-like gene DUF3435 presumably responsible for transposition, a novel gene containing DUF3723 domain, ferric reductases (FREs), patatin-like phosphatases (PLPs), and NOD-like receptors (NLRs). However, they comprise only a few copies in the host genomes (~5 copies at most), have a large variation in size (from 27 to 393 kb), and contain accessory genes (e.g., metal tolerance genes in *HEPHAESTUS*, meiotic drive genes in *Spok* block, and virulence genes in *ToxA*), providing specific traits to the hosts. Thus, *Starship* elements are akin to bacterial integrative and conjugative elements (ICEs) (52) and are suggested to play key roles in horizontal gene transfer of beneficial genes (49–51). This highly contrasts with *Teratorn,* in that it comprises a large number of copies (~40 copies) in the medaka genome and acts as a selfish genetic element. As such, further research is expected to provide new examples of giant TEs in the near future.

### The life cycle of *Teratorn*: a new life form of a herpesvirus?

A second unique character of *Teratorn* is that it exists as a chromosomally-integrated form, which is quite different from other herpesviruses. Generally, herpesviruses establish latent infection after entry into host cells, maintaining their genomes as an episomal form in the nucleus, and recurrently activate under some circumstances (53) (Figure 2D, left). Thus, chromosomal integration is not a general phenomenon for most herpesviruses. To our knowledge, among the >100 herpesvirus species reported so far, there are only two chromosomally-integrated herpesviruses other than *Teratorn*, human herpesvirus 6 (HHV-6) (54) and tarsier endogenous herpesvirus (55). Both of them belong to the *Betaherpesvirinae* subfamily of the *Herpesviridae* family, and are integrated into the telomeric region of the host chromosome through their own telomeric repeats (TMRs) at their termini (54, 56). However, it remains unclear whether these chromosomally-integrated herpesviruses are genuine genomic parasites. Tarsier endogenous herpesvirus is thought to be an already fossilized endogenous viral element (EVE), since nearly all genes have lost coding capacity accumulating deleterious mutations (55). On the other hand, chromosomally-integrated HHV-6 is estimated to exist in approximately 1% of the human population (56), and horizontal transfer is still the major way of HHV-6 transmission. In contrast, *Teratorn* exists in the genomes of several medaka-related species retaining viral genes as intact forms, and the phylogeny of *Teratorn* is the same as that of host species, suggesting long-term vertical inheritance among the genus *Oryzias* (19). We reason that chromosomal integration is an adaptive consequence to escape from host immune systems and ensure stable, vertical transmission of their progenies across host generations. Herpesviruses are successful pathogens in establishing long-term infection without chromosomal integration (53). However, since virus molecules can be detected by pathogen recognition receptors (PRRs) evoking the host immune system, herpesviruses need to take various strategies to overcome them, especially during viral replication (57). *piggyBac* acquisition of the chromosomal integration system could provide an alternative way of transmission (vertical inheritance), in addition to infection.

What is the entire life cycle of *Teratorn*? Existence of several medaka spontaneous mutants caused by *Teratorn* insertion, as well as polymorphism in integration sites among individuals of Hd-rR medaka inbred strain, indicated that it behaves at least as an active transposon *in vivo*. However, the presence of a set of intact herpesvirus-related genes suggests that those genes are also utilized for the propagation of *Teratorn*. In HHV-6, a chromosomally-integrated form of the virus was suggested to be reactivated in some situations (58, 59), accompanied by the formation of circular viral DNA molecules *via* excision of the telomeric t-loop (56, 60). It would be intriguing if *Teratorn* also undergoes similar processes during reactivation; excision mediated by the *piggyBac*-like transposase, formation of a circular form of DNA, followed by genome replication and virion formation (Figure 2D, right). However, we have not been successful in identifying virions or virus-like particles derived from *Teratorn* in medaka. Treatment of medaka fibroblast with 5′-azacytidine, a DNA methylation inhibitor, only caused moderate upregulation of viral genes, and we failed to detect any virus particles by electron microscopy or viral proteins by western blot under this condition (19). This could be due to the robust silencing of *Teratorn* by epigenetic mechanisms (61), since transposon silencing generally involves multiple mechanisms, such as DNA methylation, repressive histone modifications, KRAB zinc-finger proteins, and small RNAs (62, 63). Thus, further experiments are necessary to uncover the repression mechanisms and unlock them to fully reactivate *Teratorn*.

### Widespread distribution of *Teratorn*-like elements in teleost; generality of *piggyBac*-herpesvirus fusion

**Figure 3:**
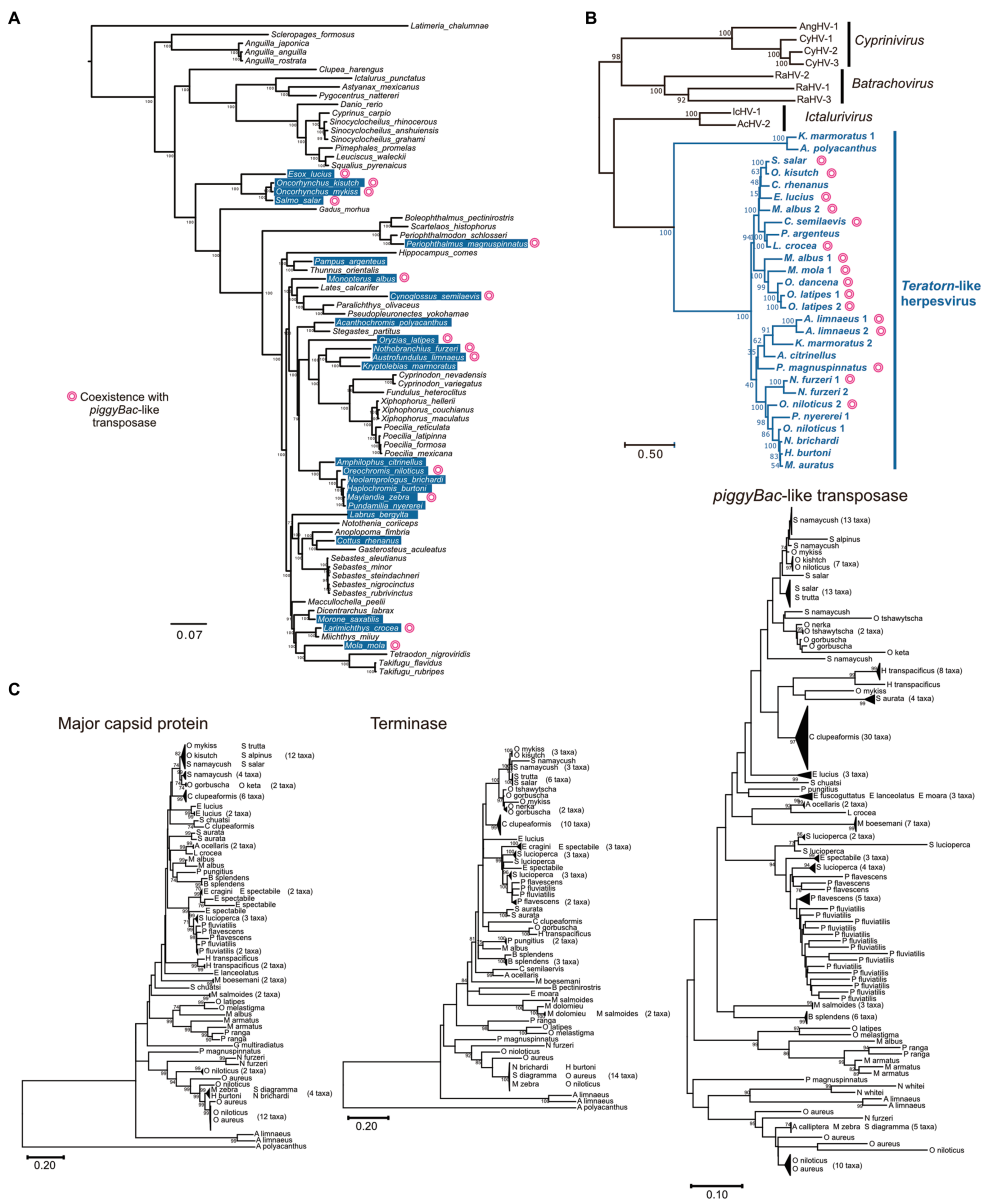
Widespread distribution of *Teratorn*-like herpesviruses in teleosts. **(A)** Distribution of *Teratorn*-like herpesviruses in the genomes of teleost fish species. Species that seem to contain *Teratorn*-like herpesvirus are highlighted in blue (greater than nine of the 13 herpesvirus core genes, tblastn *E*-value <10^−3^). The phylogenetic tree was constructed by bayesian inference, based on the concatenated nucleotide sequence of nine nuclear genes (64). Species in which *Teratorn*-like herpesviruses are adjacent to *piggyBac*-like transposase genes are marked. **(B)** Phylogeny of *Teratorn*-like herpesviruses and other alloherpesvirus species, based on the maximum-likelihood analysis. The concatenated amino acid sequences of five herpesvirus genes (major capsid protein, DNA helicase, DNA polymerase, capsid triplex protein, and DNA packaging terminase) were used. **(C)** Maximum-likelihood trees based on the nucleotide sequences of major capsid protein, DNA packaging terminase, and *piggyBac*-like transposase genes inside re-screened *Teratorn*-like herpesvirus sequences. General time reversible model was used as a substitution model, considering evolutionary rate differences among sites by discrete Gamma distribution with five categories. Total of 2,878 positions (major capsid protein), 756 positions (DNA packaging terminase), and 558 positions (*piggyBac*-like transposase) were used in the final dataset. Nodes of phylogenetic trees were collapsed if the sequence similarity was greater than 98% for major capsid protein and terminase, and greater than 96% for *piggyBac*-like transposase. Non-collapsed trees are shown in Supplementary Figure 1. The scale bars represent the number of substitutions per site.

There was an intriguing question as to whether transposon-herpesvirus fusion is a rare accidental event or a general phenomenon in nature. We previously performed a comprehensive survey of *Teratorn*-like sequences against a publicly available vertebrate genome dataset (Tblastn search of 13 herpesvirus core genes) and identified *Teratorn*-like elements in at least 22 of the 77 teleost fish species (Figure 3A) (20). In contrast, we did not get any significant hits against other vertebrate genomes, indicating that *Teratorn*-like elements were distributed only in teleosts. To date, there have been four genera in *Alloherpesviridae*; *Batrachovirus*, *Cyprinivirus*, *Ictalurivirus*, and *Salmonivirus* (65). However, phylogenetic analysis showed that *Teratorn*-like elements are quite distantly related to all those genera and form a single cluster among *Alloherpesviridae* (Figure 3B). Thus, we proposed that *Teratorn*-like elements (hereafter call *Teratorn*-like herpesviruses) represent a new genus of alloherpesvirus, characterized by a high tendency for genomic integration. Several lines of evidence support the idea of multiple integration events among teleosts, i.e., the patchy distribution of *Teratorn*-like herpesviruses among teleost fishes, and little correlation between the phylogeny of *Teratorn*-like herpesviruses and host genomes (Figures 3A,B).

**Figure 4:**
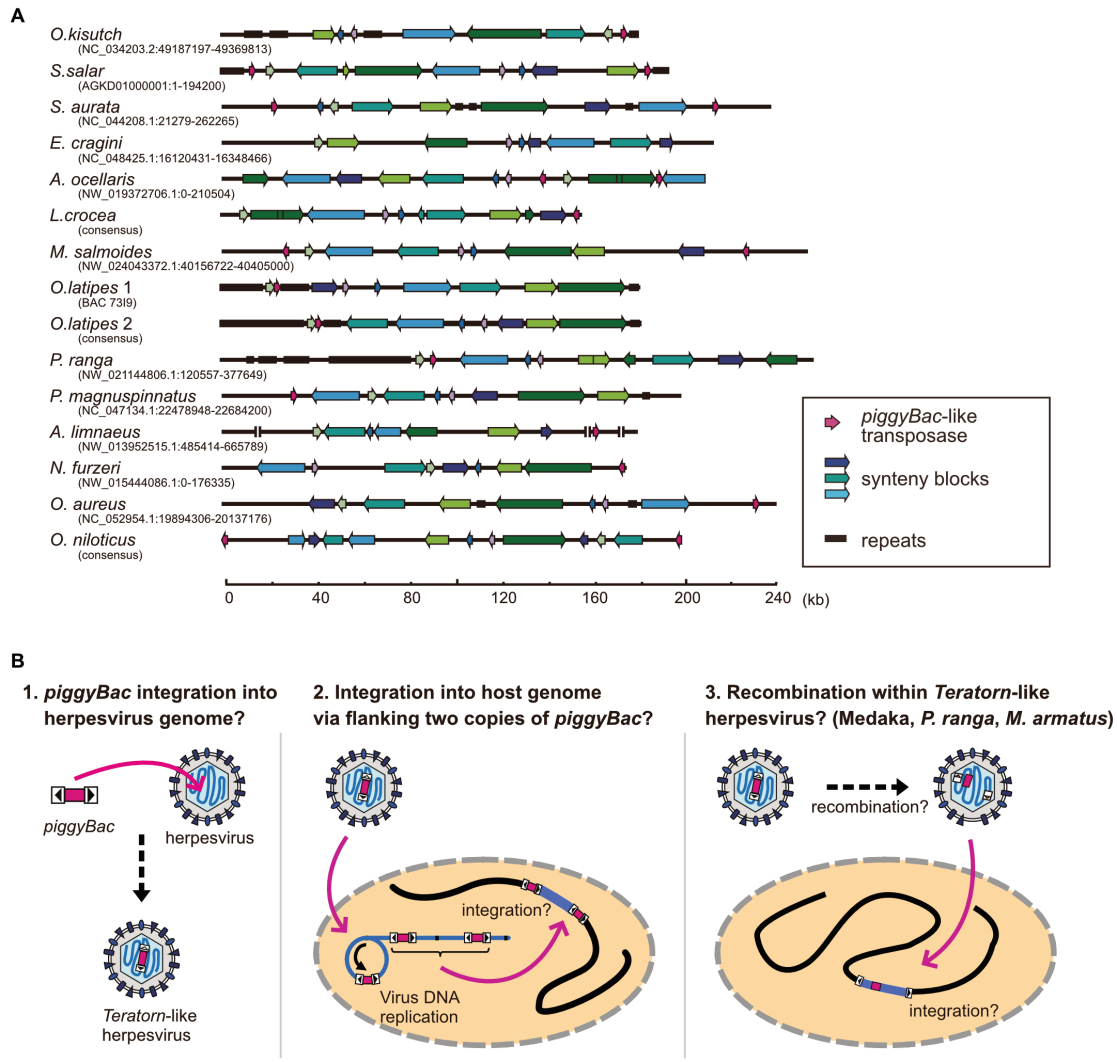
Structures of *Teratorn*-like herpesviruses and a model of the evolutionary process of *piggyBac*-herpesvirus fusion. **(A)** Structures of *Teratorn*-like herpesvirus in several teleost fish species. Conserved synteny blocks are depicted by the same colors. Magenta arrows indicate *piggyBac*-like transposase. **(B)** A model of the process of *piggyBac*-herpesvirus fusion. In this scenario, the initial event is the transposition of a *piggyBac*-like transposon into a herpesvirus genome (left). After formation of concatemeric virus DNA during replication, the internal herpesvirus genome was excised, with the two copies of *piggyBac*-like transposons as its boundary, and was integrated into the host genome (middle). A subgroup of *Teratorn*-like herpesviruses (those in medaka, *M. albus, P. ranga*, and *M. armatus*) might have undergone further recombination, resulting in *piggyBac* transposase gene located in the middle while TIRs at both ends of *Teratorn*-like herpesvirus (right).

We previously found that at least 9 of the 22 *Teratorn*-like herpesviruses have a configuration of *piggyBac*-herpesvirus fusion, and suggested the co-evolution of *piggyBac* and herpesvirus sequences, based on the same topology of phylogenetic trees between them (20). We reperformed a comprehensive BLAST search of *Teratorn*-like herpesviruses against publicly available teleost genomes (NCBI RefSeq Representative Genomes). This time we identified *Teratorn*-like herpesviruses in at least 53 of the 138 teleost fish species (tblastn, e-value <1e-05, more than five of the 15 herpesvirus genes). Importantly, we found that *piggyBac*-like transposons exist close to or inside *Teratorn*-like herpesviruses in at least 42 of the 53 species (Table 1; Supplementary Table S1), indicating that fusion with *piggyBac*-like transposon is common for *Teratorn*-like herpesviruses. Furthermore, the topology of the phylogenetic tree of *piggyBac*-like transposase genes inside *Teratorn*-like herpesviruses is again nearly identical to that of herpesvirus genes (Figure 3C; Supplementary Figure S1). These data strongly suggest that *piggyBac*-like transposon and herpesvirus genome have behaved as a single unit for a long evolutionary timescale and that the fusion has allowed *Teratorn*-like herpesviruses for widespread habitation among teleost genomes. Structures of the representative *Teratorn*-like herpesviruses are shown in Figure 4A.

**Table 1:** Tblastn of alloherpesvirus core genes of medaka *Teratorn* against teleost genomes.

	Pol	Hel	Pri	MCP	Triplex	Env	Term	Pro	ORF34	ORF37	ORF44	ORF54	ORF56	ORF60	ORF64	No. of genes (tblastn, e-value < 1e-05)	*piggyBac*-fusion?
*Acanthochromis polyacanthus*	●		●	●			●			●			●	●		7	
*Amphiprion ocellaris*	●	●	●	●	●	●	●	●	●	●	●	●	●	●	●	15	*piggyBac* locates adjacent to *Teratorn*-like herpesvirus
*Austrofundulus limnaeus*	●	●	●	●	●	●	●	●	●	●	●	●	●	●	●	15	*piggyBac* locates adjacent to *Teratorn*-like herpesvirus
*Betta splendens*	●	●	●	●	●	●	●	●	●	●	●	●	●	●	●	15	*piggyBac* locates adjacent to *Teratorn*-like herpesvirus
*Coregonus clupeaformis*	●	●	●	●	●	●	●	●	●	●	●	●	●	●	●	15	*piggyBac* locates adjacent to *Teratorn*-like herpesvirus
*Cynoglossus semilaevis*	●		●	●	●		●	●	●	●	●	●	●	●	●	13	*piggyBac* locates adjacent to *Teratorn*-like herpesvirus
*Epinephelus fuscoguttatus*	●		●									●	●	●		5	*piggyBac* locates adjacent to *Teratorn*-like herpesvirus
*Epinephelus lanceolatus*		●		●	●	●		●	●	●	●	●		●		10	*piggyBac* locates adjacent to *Teratorn*-like herpesvirus
*Epinephelus moara*	●	●	●	●	●	●	●	●	●	●	●		●	●	●	14	*piggyBac* locates adjacent to *Teratorn*-like herpesvirus
*Esox lucius*	●	●		●	●	●	●	●	●	●	●	●	●	●	●	14	*piggyBac* locates adjacent to *Teratorn*-like herpesvirus
*Etheostoma cragini*	●	●	●	●	●	●	●	●	●	●	●	●	●	●	●	15	
*Etheostoma spectabile*	●	●	●	●	●	●	●	●	●	●	●	●	●	●	●	15	*piggyBac* locates adjacent to *Teratorn*-like herpesvirus
*Girardinichthys multiradiatus*	●	●	●	●		●	●			●	●	●	●	●	●	12	
*Haplochromis burtoni*	●	●	●	●	●	●	●		●	●	●	●	●	●	●	14	
*Hypomesus transpacificus*	●	●	●	●	●	●	●	●	●	●	●	●	●	●	●	15	*piggyBac* locates adjacent to *Teratorn*-like herpesvirus
*Labrus bergylta*	●				●			●		●			●			5	
*Larimichthys crocea*	●	●	●	●	●	●	●	●	●	●	●	●	●	●	●	15	**fusion confirmed** (*piggyBac* locates adjacent to *Teratorn*-like herpesvirus)
*Mastacembelus armatus*	●	●		●	●	●	●	●	●	●	●	●	●	●		13	*piggyBac* locates inside *Teratorn*-like herpesvirus
*Maylandia zebra*	●	●	●	●	●	●	●	●	●	●	●	●	●	●	●	15	*piggyBac* locates at both ends of *Teratorn*-like herpesvirus
*Melanotaenia boesemani*	●	●	●	●	●	●	●	●	●	●	●	●	●	●	●	15	*piggyBac* locates adjacent to *Teratorn*-like virus
*Micropterus dolomieu*			●	●			●		●			●			●	6	
*Micropterus salmoides*	●	●	●	●	●	●	●	●	●	●	●	●	●	●	●	15	*piggyBac* locates at both ends of *Teratorn*-like virus
*Monopterus albus*	●	●	●	●	●	●	●	●	●	●	●	●	●	●	●	15	*piggyBac* locates inside *Teratorn*-like virus
*Morone saxatilis*				●	●	●						●		●		5	
*Nematolebias whitei*	●	●			●		●						●	●	●	7	*piggyBac* locates adjacent to *Teratorn*-like herpesvirus
*Neolamprologus brichardi*	●	●	●	●	●	●	●	●	●	●	●	●	●	●	●	15	
*Nothobranchius furzeri*	●	●	●	●	●	●	●	●	●	●	●	●	●	●	●	15	*piggyBac* locates adjacent to *Teratorn*-like herpesvirus
*Oncorhynchus gorbuscha*	●	●	●	●	●	●	●	●	●	●	●	●	●	●	●	15	*piggyBac* locates at both ends of *Teratorn*-like herpesvirus
*Oncorhynchus keta*	●	●		●	●	●	●	●	●	●	●	●	●	●	●	14	*piggyBac* locates adjacent to *Teratorn*-like herpesvirus
*Oncorhynchus kisutch*	●	●	●	●	●	●	●	●	●	●	●	●	●	●	●	15	*piggyBac* locates adjacent to *Teratorn*-like herpesvirus
*Oncorhynchus mykiss*	●	●	●	●	●	●	●	●	●	●	●	●	●	●	●	15	*piggyBac* locates adjacent to *Teratorn*-like herpesvirus
*Oncorhynchus nerka*	●	●	●		●	●	●	●				●	●	●	●	11	*piggyBac* locates adjacent to *Teratorn*-like herpesvirus
*Oncorhynchus tshawytscha*	●	●	●	●	●	●	●	●	●	●	●	●	●	●	●	15	*piggyBac* locates adjacent to *Teratorn*-like herpesvirus
*Oreochromis aureus*	●	●	●	●	●	●	●	●	●	●	●	●	●	●	●	15	*piggyBac* locates adjacent to *Teratorn*-like herpesvirus
*Oreochromis niloticus*	●	●	●	●	●	●	●	●	●	●	●	●	●	●	●	15	**fusion confirmed** (*piggyBac* locates at both ends of *Teratorn*-like herpesvirus)
*Oryzias latipes*	●	●	●	●	●	●	●	●	●	●	●	●	●	●	●	15	**fusion confirmed** (*piggyBac* locates inside *Teratorn*-like herpesvirus)
*Oryzias melastigma*	●	●	●	●	●	●	●	●	●	●	●	●	●	●	●	15	**fusion confirmed** (*piggyBac* locates inside *Teratorn*-like herpesvirus)
*Parambassis ranga*	●	●	●	●	●	●	●	●	●	●	●	●	●	●	●	15	*piggyBac* locates inside *Teratorn*-like herpesvirus
*Perca flavescens*	●	●	●	●	●	●	●	●	●	●	●	●	●	●	●	15	*piggyBac* locates adjacent to *Teratorn*-like herpesvirus
*Perca fluviatilis*	●	●	●	●	●	●	●	●	●	●	●	●	●	●	●	15	*piggyBac* locates adjacent to *Teratorn*-like herpesvirus
*Periophthalmus magnuspinnatus*	●	●	●	●	●	●	●	●	●	●	●	●	●	●	●	15	*piggyBac* locates adjacent to *Teratorn*-like herpesvirus
*Pundamilia nyererei*	●	●	●	●	●	●	●	●	●	●	●	●	●	●	●	15	
*Pungitius pungitius*	●	●	●	●	●	●	●	●	●	●	●	●	●	●	●	15	*piggyBac* locates adjacent to *Teratorn*-like herpesvirus
*Salmo salar*	●	●	●	●	●	●	●	●	●	●	●	●	●	●	●	15	*piggyBac* locates adjacent to *Teratorn*-like herpesvirus
*Salmo trutta*	●	●	●	●	●	●	●	●	●	●	●	●	●	●	●	15	*piggyBac* locates adjacent to *Teratorn*-like herpesvirus
*Salvelinus alpinus*	●	●		●	●	●	●	●		●		●	●	●	●	12	*piggyBac* locates adjacent to *Teratorn*-like herpesvirus
*Salvelinus namaycush*	●	●	●	●	●	●	●	●	●	●	●	●	●	●	●	15	*piggyBac* locates adjacent to *Teratorn*-like herpesvirus
*Sander lucioperca*	●	●	●	●	●	●	●	●	●	●	●	●	●	●	●	15	*piggyBac* locates adjacent to *Teratorn*-like herpesvirus
*Scophthalmus maximus*	●					●	●				●		●		●	6	
*Simochromis diagramma*	●	●	●	●	●	●	●	●	●	●		●	●	●	●	14	*piggyBac* locates adjacent to *Teratorn*-like herpesvirus
*Siniperca chuatsi*	●		●	●		●	●		●	●	●	●	●	●		11	*piggyBac* locates adjacent to *Teratorn*-like herpesvirus
*Sparus aurata*	●	●	●	●	●	●	●	●	●	●	●	●	●	●	●	15	*piggyBac* locates at both ends of *Teratorn*-like herpesvirus
*Xiphias gladius*		●	●		●	●		●		●	●	●		●		9	

### Process of *piggyBac*-herpesvirus fusion; *piggyBac* transposition into herpesvirus genome, followed by recombination?

The underlying mechanism for the fusion of an ancestral herpesvirus and a *piggyBac*-like transposon to create *Teratorn*-like herpesvirus is largely unknown, but based on the configuration of *Teratorn*-like herpesviruses in teleosts, we propose the following scenario. The first event of the fusion would be that a *piggyBac* transposon was jumped from the host genome into the herpesvirus genome during viral infection (Figure 4B left). Indeed, previous studies reported that TEs can transpose into the genomes of dsDNA viruses of various families, such as Baculoviruses (66–69), Poxviruses (70), Iridoviruses (69), and Pandraviruses (71), possibly whereby acting as vectors of horizontal transposon transfer across species (72). Although transposon insertion has not yet been reported so far for herpesviruses, it could occur, from the host genome into the virus DNA, since herpesviruses usually establish a latent infection with their genomic DNA floating in the nucleus of host cells. Once DNA transposon is transposed into the herpesvirus DNA, integration of the whole virus genome into the host chromosome is theoretically possible. During herpesvirus replication, viral genomic DNA becomes circular and DNA synthesis proceeds in a rolling-circle manner, giving rise to concatenated virus DNA (73). Thus, a single unit of viral genomic DNA, flanked by two copies of *piggyBac*-like DNA transposons, could be integrated into host genomes *via* transposition (Figure 4B middle). This scenario is supported by the fact that the majority of *Teratorn*-like herpesviruses contain *piggyBac*-like transposon at their termini (Figure 4A; Supplementary Table S1). However, a clade of *Teratorn*-like herpesviruses (those in medaka, *M. albus, P. ranga*, and *M. armatus*) must have undergone further recombination, resulting in the *piggyBac* transposase gene located in the middle while TIRs at both ends of *Teratorn* (Figures 4A,B right). Of course, to test the above scenario, a further genomic survey of *Teratorn*-like herpesviruses in teleost genomes, as well as identification of the exogenous form of *Teratorn*-like herpesviruses, will definitely be required.

### Recombination as a key driver of life cycle shift of MGEs and viruses?

Due to the lack of chromosomal integration step in the life cycles, it was generally thought that nearly all viruses except for retroviruses have lost propagation capacity after genomic integration (14). Indeed, except for ERVs, there have been only a few reports of EVEs that have increased the copy number inside host genomes. Such examples are endogenous pararetroviruses and some single-stranded DNA viruses. The former constitutes up to ~1% of the genome of some plants (74), although the mechanism underlying chromosomal integration and propagation is unknown. The latter is present in tens to one thousand copies in some species (e.g., geminiviruses in plants and fungi, circoviruses and parvoviruses in animals) and is thought to integrate *via* RC-Rep endonuclease, an enzyme that functions in replication of virus DNA, although not experimentally confirmed (75–77). Thus, we propose that *Teratorn*-like herpesviruses are the first endogenous non-retroviral element that shifted into the intragenomic lifestyle to promote propagation in host genomes. Recently, some giant (up to ~2000 kb) dsDNA viruses belonging to Mimiviruses and Phycodnaviruses were found to be integrated into the genomes of diverse green algae species (78). Although the mechanism of integration is unknown again, this study raised the possibility that integration of large dsDNA viruses into host genomes widely occurs, some of which might integrate and propagate in the host genomes with the help of DNA transposons.

**Figure 5:**
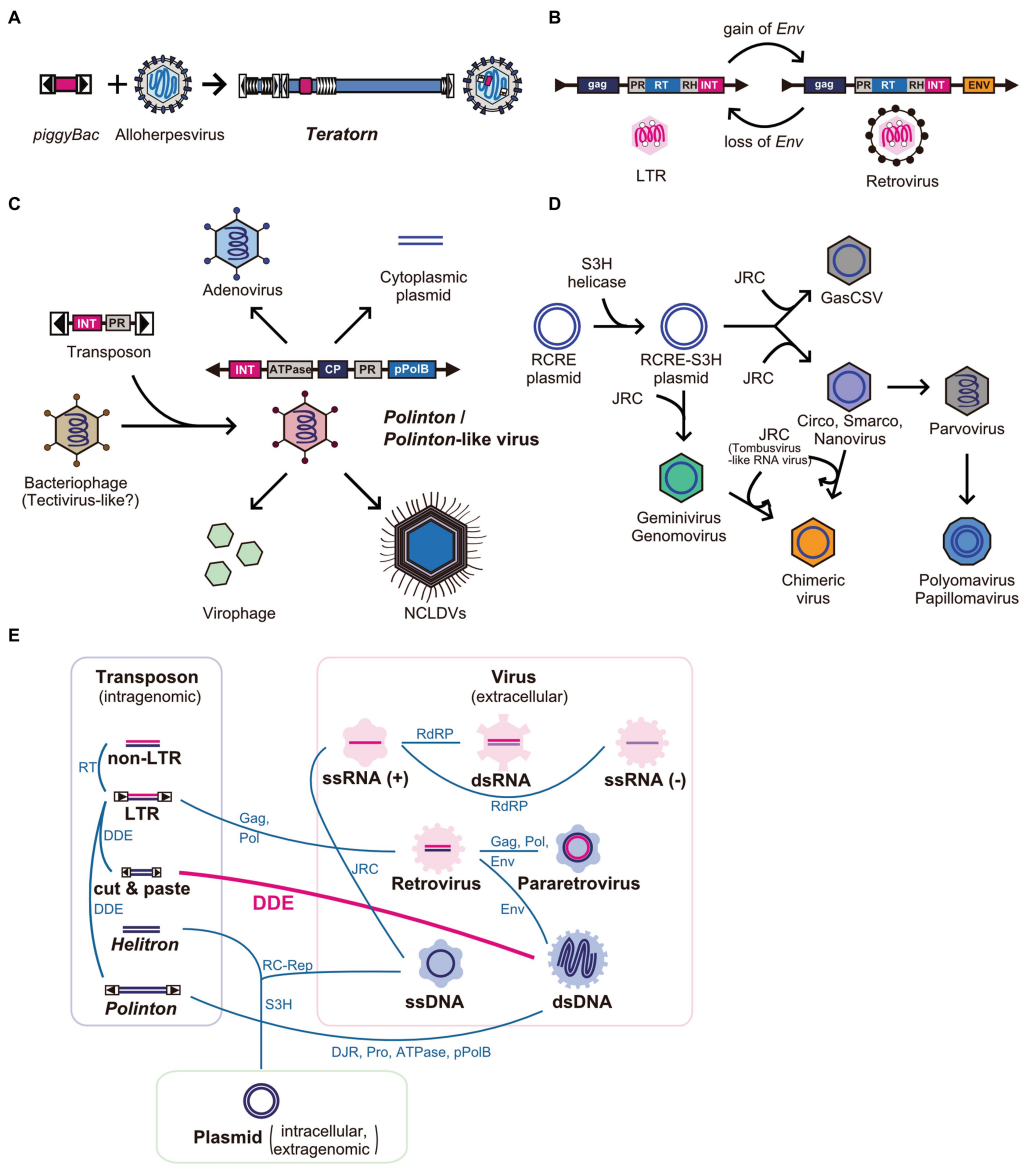
Network-like evolutionary relationships in eukaryotic mobile genetic elements and viruses. **(A–D)** Examples of the emergence of novel mobile genetic elements by recombination between different class of elements. **(A)** *Teratorn* **(B)** LTR retrotransposons and retroviruses **(C)** *Polintons* and several dsDNA viruses, adapted from (79) **(D)** ssDNA viruses, adapted from (80). Abbreviations; PR, protease; RH, RNA helicase; RT, reverse-transcriptase; Env, envelope protein; INT, retrovirus-like integrase; CP, capsid protein; pPolB, protein-primed family B DNA polymerase; NCLDV, nucleocytoplasmic large DNA virus; RCRE, rolling-circle replication endonuclease; JRC, jerry-roll capsid. **(E)** Network-like evolutionary relationships in eukaryotic mobile genetic elements and viruses. Evolutionary relationships between different classes of mobile elements are connected by lines. Shared genes are represented next to the lines. Abbreviations; ATPase, DNA packaging ATPase; DDE, DDE transposase; DJR, double jelly-roll capsid; Env, envelope protein; Gag, group-specific antigen; JRC, jelly-roll capsid; pPolB, protein-primed family B DNA polymerase; Pro, Ulp1-like cysteine protease; RC-Rep, rolling-circle replication initiation endonuclease; RdRP, RNA-dependent RNA polymerase; RT, reverse-transcriptase; S3H, superfamily 3 helicase.

So far, *Teratorn* is the first example of the fusion between cut-and-paste DNA transposon and DNA virus (Figure 5A). However, there are several examples of the fusion of two or more distinct mobile elements in the network of eukaryotic MGEs and viruses. The most famous and classical example is the transition from LTR retrotransposons to retroviruses (14, 18) and vice versa (81) (Figure 5B). The former transition is thought to be mediated by the gain of envelope genes from other viruses such as baculovirus and herpesvirus (18), while the latter could be a result of chromosomal integration into germline cells followed by propagation inside host genomes (81). Recent phylogenetic analyses suggest that such recombination events are rather common for all MGEs and viruses and have contributed to their diversification (82). The first example is *Polintons* (also known as *Mavericks*), a group of replicative large DNA transposons (10–40 kb) widely distributed in eukaryotes. *Polintons* were initially regarded as transposons but were later found to encode two capsid protein genes (double jerry roll major capsid protein (DJR-MCP) and minor capsid protein) (83), suggesting that they are *bona fide* viruses. Recent phylogenetic studies suggested that *Polintons* have evolutionary links with various viruses and MGEs (e.g., adenoviruses, nucleocytoplasmic large DNA viruses (NCLDVs), virophages, *Polinton*-like viruses, linear plasmids, and tectivirus-like bacteriophages), characterized by the shared genes between groups (e.g., DJR-MCP, minor capsid protein, protein-primed DNA polymerase (pPolB), Ulp1-like cysteine protease, A32-like genome packaging ATPase, and retrovirus-like integrase (RVE-INT)) (79, 83–86). Based on the phylogenetic analyses, the following scenario was proposed for the evolution of those viruses and MGEs. First, *Polinton* emerged from a tectivirus-like bacteriophage *via* the acquisition of a retrovirus-like integrase gene and cysteine protease gene from a certain DNA transposon related to *Ginger 1*, at the emergence of eukaryotes. Then, some of them returned to virus life forms, concurrent with the loss of integrase genes and/or exchange of DNA polymerase genes, which enabled the expansion of their genomes to form a large part of current eukaryotic DNA viruses such as adenoviruses and NCLDVs (79, 83–85) (Figure 5C). However, more recently, comprehensive surveys of *Polinton*-like viruses against metagenomic samples were performed and found that *Polintons* are polyphyletic among *Polinton*-like viruses. This suggests that the evolution of this group of elements is more complex than previously thought, and the transition from a transposon to a virus and vice versa is massively ongoing for this group of elements (85, 87). Another example is the evolution of ssDNA viruses, which also seemed to be driven by multiple recombination events between different classes of elements. In this scenario, eukaryotic ssDNA is thought to originate from rolling-circle replicating plasmids by the acquisition of single jelly-roll capsid protein-encoding genes from ssRNA viruses (15, 80) (Figure 5D). Such recombination events are suggested to be ongoing even now. This is supported by the finding of a novel group of ssDNA viruses named chimeric viruses (CHIVs), which are created by the acquisition of a capsid gene from another family of ssRNA viruses (tombusviruses) by several families of ssDNA viruses (geminiviruses, circoviruses, and nanoviruses) (88) (Figure 5D). The most extreme example of recombination is bidnavirus, a single-stranded DNA virus, which is composed of sequences derived from four different families of viruses and transposons [*Polinton*-derived DNA polymerase gene (pPolB), parvovirus-derived capsid gene, and additional two genes derived from reovirus and baculovirus (89)]. Thus, the recombination and fusion between mobile elements of distinct classes occur more frequently than previously thought, in the process of the diversification of MGEs and viruses (Figure 5E). So far, *Teratorn* has provided the most concrete evidence for this concept. Further identification of novel and peculiar MGEs and viruses will provide insights into mechanisms underlying their diversification and evolution.

## Conclusion

In summary, *Teratorn* has the following unique features; (I) It is very large (~180 kb long) and active transposon, (II) created by a fusion of a *piggyBac*-like DNA transposon and the whole genome of a herpesvirus, (III) together with related herpesviruses, forms a new genus among *Alloherpesviridae*, characterized by a high tendency for intragenomic propagation. Thus, *Teratorn* provides new insights into not only transposon and herpesvirus biology but also into the mechanisms of how novel MGEs and viruses emerge. However, many questions remain unanswered. For example, the genuine amplification mechanism of *Teratorn* is still unclear. Secondly, the impacts of *Teratorn* on host species are not fully investigated. Given its large size, *Teratorn* insertion could alter the 3D chromatin conformation around its inserted region, which frequently impacts gene regulation and causes serious phenotypes. Thirdly, uncovering the arms race between *Teratorn* and host defense systems (e.g., immune systems and epigenetic silencing) is of interest. Lastly, the evolutionary process of *piggyBac*-herpesvirus fusion, and the generality of DNA transposon-DNA virus fusion is unclear. Answering the above questions will require combinatorial approaches covering virology, genome science, and evolutionary biology.

## Methods

### Search for *Teratorn*–like herpesviruses in teleost fish species

A tblastn search of 15 herpesvirus genes of *Teratorn* (DNA polymerase, DNA helicase, primase, ATPase subunit of terminase, major capsid protein, membrane glycoprotein, capsid triplex protein, capsid maturation protease, ORF34, ORF37, ORF44, ORF54, ORF56, ORF60, ORF64) was carried out against all available teleost genomes (NCBI RefSeq Representative Genomes, 138 species in total) with default parameters. Sequences of *Teratorn*-like herpesvirus were obtained as follows; First, locations of the 15 herpesvirus genes were identified by tblastn. After merging the genomic loci of each herpesvirus gene which are within 40 kb of one another, sequences of the defined region and the flanking 40 kb region were extracted from the draft genomes using BEDtool (90). The list of *Teratorn*-like herpesviruses is shown in Supplementary Table S1.

### Phylogenetic analysis

Nucleotide sequences of major capsid protein, DNA packaging terminase, and *piggyBac*-like transposase were obtained by tblastn search against *Teratorn*-like herpesvirus sequences. Nucleotide alignments were constructed using MUSCLE in MEGA11 (91), followed by removal of poorly aligned regions using trimAl (92) with a –strict option. Maximum-likelihood analysis was performed for each element using MEGA11 with 100 bootstraps (general time reversible model, discrete gamma distribution with five rate categories). All positions with less than 70% site coverage were eliminated; i.e., fewer than 30% alignment gaps, missing data, and ambiguous bases were allowed at any position (partial deletion option). Multiple alignment data was attached in Supplementary Text 1.

## Author contributions

YI prepared the figures and performed a search of *Teratorn*-like herpesviruses and phylogenetic analyses. YI and HT wrote the manuscript. All authors contributed to the article and approved the submitted version.

## Conflict of interest

The authors declare that the research was conducted in the absence of any commercial or financial relationships that could be construed as a potential conflict of interest.

## Publisher’s note

All claims expressed in this article are solely those of the authors and do not necessarily represent those of their affiliated organizations, or those of the publisher, the editors and the reviewers. Any product that may be evaluated in this article, or claim that may be made by its manufacturer, is not guaranteed or endorsed by the publisher.
