# Supplementary figures and images for: Teratorn and its relatives – a cross-point of distinct mobile elements, transposons and viruses
Source: Front Vet Sci. 2023 Apr 28;10:1158023. doi: 10.3389/fvets.2023.1158023 (PMC10175614; doi:10.3389/fvets.2023.1158023)

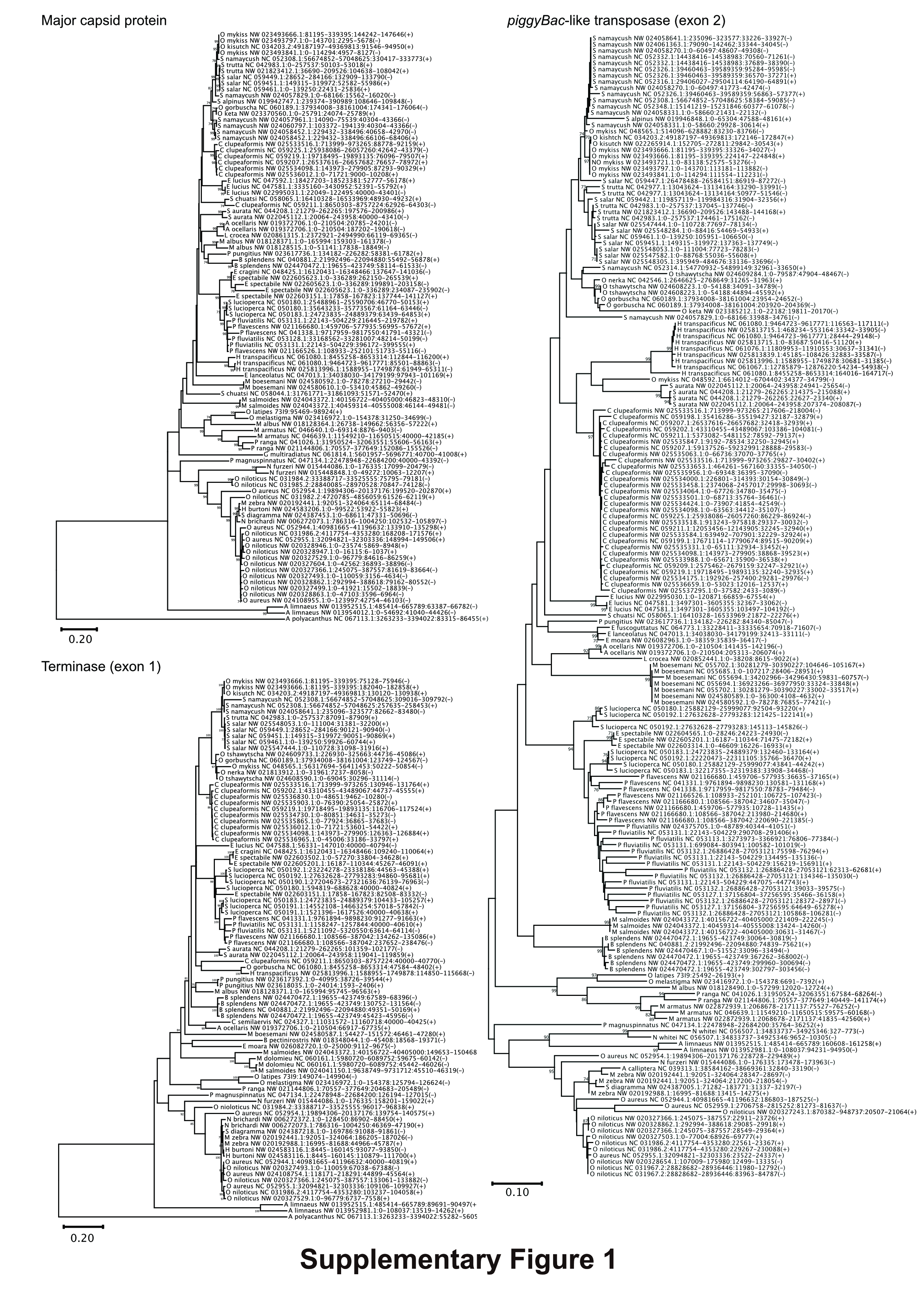

Supplement: Supplementary file 3 [file Image_1.TIFF]
